# Half of the mothers had poor delivery referral practices in public hospitals of Bahir Dar City Northwest, Ethiopia

**DOI:** 10.3389/fpubh.2025.1452254

**Published:** 2025-02-26

**Authors:** Amare Fenta Abebe, Desta Debalkie Atnafu, Habtamu Alganeh Guadie

**Affiliations:** ^1^Feleghiwot Referral Hospital, Bahir Dar, Ethiopia; ^2^Department of Health Systems Management and Health Economics, School of Public Health, College of Medicine and Health Sciences, Bahir Dar University, Bahir Dar, Ethiopia

**Keywords:** referral practice, laboring mother, poor practice, Bahir Dar City, Ethiopia

## Abstract

**Background:**

Policy makers and stakeholders may benefit from understanding maternal delivery referral practices as they develop efficient mechanisms to implement appropriate referral linkage. However, the practice of maternal delivery referral is not well known. This study aims to assess the maternal referral practices and associated factors among laboring mothers referred to public hospitals of Bahir Dar City, Northwest, Ethiopia.

**Method:**

In the hospitals of the city of Bahir Dar, a facility-based cross-sectional survey was carried out from March 1 to March 30, 2021. A total of 358 mothers who came by referral to give birth at the public hospitals in Bahir Dar were interviewed using a pre-tested questionnaire that was presented by an interviewer. Data was coded, and inputted to Epi-data version 3.1 software, and after being transferred, analyzed using SPSS version 25. The associated factors linked to poor maternal referral practices were identified using bi-variable and multivariable logistic regressions. The p-value cutoff of 0.05 was ultimately determined to be statistically significant.

**Results:**

A total of 353 study participants took part in the study. The level of poor maternal referral practice was 52.7% (95% CI 47%, 58%). The mean age of the respondents was 26.73 (± 5.45) years. Twenty eight percent of the mothers were illiterate. Unable to read and write (AOR = 2.38, 95%CI: 1.15, 4.94), read and write only (AOR = 6.59, 95%CI: 2.53, 17.17), monthly income < 1,527 birr (AOR = 4.55, 95%CI: 1.91, 10.84), monthly income between 1,527 and 3,000 birr (AOR = 4.29, 95%CI: 1.76, 10.50), and monthly income between 3,001 and 5,305 birr (AOR = 3.73, 95%CI: 1.49, 9.33), referred from referral hospitals (AOR = 4.63, 95%CI: 1.94, 11.07), gave birth via cesarean section (AOR = 2.06, 95%CI: 1.22, 3.47), gave birth via assisted delivery (AOR = 4.77, 95%CI: 1.64, 13.91), and time spent more than 1 h to arrive to Bahir Dar City public hospitals (AOR = 2.15, 95%CI: 1.07, 4.34) were significantly associated with poor maternal referral practice.

**Conclusion:**

Poor maternal referral practices were widespread. The use of maternal referrals was influenced by obstetric, social, environmental, and demographic factors. The poor maternal referral practices during labor should receive the most attention from mothers who have low monthly incomes and do not attend formal education.

## Background

A referral is a two-way communication process in which a healthcare professional at one level of the healthcare system requests the assistance of a better or differently resourced facility at the same or higher level to assist in, or take over, the management of the client's case because they lack the resources (drugs, equipment, skills) to do so (1). Initiating a referral can shorten treatment delays, prevent system overload, and allow the use of expertise at advanced clinical hubs by communicating the reasons for the recommendation with the receiving hospital.

The goal of the maternity referral system is to identify pregnant women who are at a high risk of obstetric difficulties and refer them to more specialized delivery care at a higher level. Risk screening throughout pregnancy is part of this strategy.

Evidence from the United States (USA) revealed that 19.8% of referrals lacked a specific justification. Additionally, just 6.4% of referrals were ruled inappropriate (2).

Only 3% of sick children who were seen at a referral hospital in Tanzania, according to a research, were referred (3). Similar to this, a research conducted in Ghana found that only 11% of the children hospitalized to the referral facility were referred, and only one outpatient department (OPD) care seeker in every 34 OPD care seekers (3%) seen at a referral institution was referred (4).

According to the Kenya National Bureau of Statistics, 2011 data showed that referral forms used to refer patients varied by province. The higher proportion of this referral was seen in western (78%), and in Nairobi providences (73%). However, the lower was recorded in rift valley (38%) and North Eastern provinces [28%; (5)].

Evidence suggested that the referral system may be impacted by lack of knowledge about referrals, inadequate information provided by doctors who refer patients, delayed or poor quality of consultations with medical professionals. The severity of the sickness, the requirement for permission to travel to the clinic and pay associated expenditures, balancing child care obligations, the perceived quality of hospital care, and the communication skills of health personnel are all significant variables, according to the studies (6, 7).

Results showed that information between medical institutions is inadequately communicated, delayed medical record transfers may result in repeated testing, and doctors have restricted authorization to prescribe certain medications, all of which have an impact on how successful the referral system is (8–11).

Inadequate obstetric transfer was the cause of nearly half of maternal deaths (48.38%; 11) between 2008 and 2013 despite the Ethiopian women's access to basic delivery care services having increased dramatically by ~48% (10-fold to 2001) by 2019. Referral practices for maternity deliveries are still up for debate (12). However, Ethiopian maternal referral linkage has little empirical support. There for, the aim of this study was to evaluate the maternal delivery referral practice and the factors that contribute to it in the public hospitals of Bahir Dar City, Ethiopia.

## Methods and materials

### Study setting and period

March 1 and March 30 of 2021, the study was carried out in the public hospitals of the city of Bahir Dar. The city, which serves as the Amhara Regional State's capital, lies 480 kilometers Northwest of Addis Ababa. There are 10 health clinics and three public hospitals in the city. Felege-hiwot Hospital, Tibebe-ghion Hospital, and Adis-alem Hospital are the public hospitals that offer delivery care services. These three public hospitals provided 1, 875 maternal delivery care services in total, according to the department of maternity reports from the preceding 3 months in the hospitals involved in this study. Of which, 131 delivered mothers were sent to Adis-alem hospitals, 953 were sent to Felege-hiwot hospitals, and 791 were sent to Tibebe-ghion hospitals.

### Study design and population

A cross-sectional study with a focus on healthcare facilities was conducted. During the time of data collection, all women who requested better delivery care at one of the three public hospitals in Bahir Dar City and were willing to participate in the study were taken into account. Women with severe medical conditions, mental health issues, or those who refused to participate in the study were not included.

### Sampling size and sampling procedure

Using a single population percentage formula, the sample size was calculated under the following presumptions: The proportion of obstetric referrals recorded in Addis Ababa was 30.3%, a 95% level of confidence and a 5% margin of error (13). The sample size was computed using a population adjustment formula and by including a 10% non-response rate because the source population, or 1, 875, was less than 10,000. As a result, the final sample size was calculated to be 358. According to proportional to size allocation, the required sample size from each public hospital was determined. Every five intervals, participants were chosen from each facility using a systematic random sample technique equal to the volume of maternal referrals to the hospitals. The sampling interval was determined using the anticipated 3-month gestational referral. The first case was chosen using a lottery system. Following the completion of the services, an exit interview was done with a total of 358 delivery women.

### Operational definitions

**Laboring mother:** is used to describe any mother who gives birth to a child and delivers the placenta, membranes, and umbilical cord to the outside world through the vagina (14).

**Maternal referral practice:** is a situation when a mother has been referred by a health professional to higher-level health care facilities and has gotten a good maternal referral practice when she responded to greater or equal to mean and above of the thirteen- referral practice assessing questions as correctly. Otherwise, she was considered as having poor maternal referral practice.

**Knowledge:** 10 questions were used to measure the knowledge of referred mothers with regard to Ethiopian healthcare referral systems. If the delivered mother answered more than the mean score of the knowledge questions, she was considered as having adequate knowledge, otherwise, she was considered as having inadequate knowledge.

**Quality of care:** In this study quality of maternal care was considered when a laboring mother gained service with respect, privacy maintained and given necessary medications as per the health professionals order.

**Data collection, studied variables and data analysis:** Structured questionnaires adapted from various studies (15–19) were used to collect the data. The survey included questions assessing socio-demographic characteristics, obstetrics-related, knowledge, environmental factors, health facility-related factors, and maternal referral practice (outcome variable). Independent translators translated the questionnaire from English to Amharic before the data collection to ensure consistency. The study's aims, data collection techniques, and the significance of the study in relation to the studied objectives were covered in a one-day training session for the four BSc midwives and the two supervisors. The responders were interviewed shortly after giving birth, just as they were getting some rest before leaving the hospital ward.

The investigators verified the consistency and completeness of the data collected. The data was coded and entered into the EPI-DATA program version 3.1 before being exported for cleaning up and analysis in SPSS version 20.0. The frequency, proportion, means, and standard deviations of descriptive statistics were computed. In order to examine the relationship between explanatory and outcome variables, a binary logistic regression model was used. For the multivariable logistic regression analysis, variables having a *p*-value of < 0.2 in the bi-variable analysis were regarded as candidates. The strength of the relationship between explanatory factors and the maternal referral practice was assessed using an adjusted odds ratio (OR) with a 95%. Variables with a *p*-value of 0.05 were considered as statistically significant predictors of maternal referral practice. The Hosmer and Lemeshow goodness of fit test was used to evaluate the model's fitness (*P* > 0.05).

### Ethical consideration

Ethical clearance for this study was obtained from the Ethical Review Board of Bahir Dar University, College of Medicine and Health Sciences. Letters of support were received from the Amhara regional public health institute. The oral consent was obtained from medical directors of each hospital. Before collecting the data, the written informed consent was obtained from delivered mothers. Anonymity and confidentiality were considered. The study adhered to tenets of the Declaration of Helsinki.

### Patient and public involvement

Patients and/or the public were not involved in the design, or conduct, or reporting, or dissemination plans of this research. The authors intend to disseminate this research to the public through social media, press releases, and media departments and websites of authors' institutions.

## Results

### Socio-demographic characteristics

A total of 353 women were enrolled in the study, and 98.6% of them responded. The mean age of respondents was 26.73 years (+ 5.45 SD). In the age range of 31–43 years, two thirds of respondents who perceived poor maternal referral practices were found. The majority of respondents lived in urban areas, including both the poor (76.4%) and those who believed that good maternal referral practices were in place (62.96%). The variables age, religion, occupation, marital status, and ethnicity did not show variation between the poor and good maternal referral practices, but maternal and husband educational status, residence, and monthly income did show a significant difference between the good perceived maternal referral practice and the poor one (Table 1).

**Table 1:** Socio-demographic characteristics of the study participants in public hospitals of Bahir Dar City, Ethiopia, 2021 (*n* = 353).

**Variables**	**Maternal delivery referral practice**		**X^2^, *P*-Value**
	**Good**	**Poor**	
**Age groups (Years);** μ = **26.73** ± **5.45 years**			
15–19	10 (41.7)	14 (58.3)	1.865, 0.393
20–30	130 (49.4)	133 (50.6)	
31–43	27 (33.3)	39 (66.7)	
**Maternal educational status**			
Unable to read and write	37 (38.1)	60 (61.9)	26.021, 0.0001^*^
Able to read and write	9 (20.9)	34 (79.1)	
Grade 1–8	28 (56)	22 (44)	
Grade 9–10	39 (55.7)	31 (44.3)	
Grade 11–12	6 (42.8)	8 (57.2)	
Diploma and above	48 (60.8)	31 (39.2)	
**Religion**			
Orthodox Christian	149 (48.4)	159 (51.6)	2.030, 0.566
Others^¥^	18 (40)	27 (60)	
**Occupation**			
Governmental employee	51 (46.8)	58 (53.2)	0.854, 0.931
Merchant	21 (50)	21 (50)	
Housewife	74 (46.3)	86 (53.7)	
Others^¥¥^	21 (50)	21 (50)	
**Marital status**			
Currently married	148 (46.4)	171 (53.6)	3.145, 0.370
Currently unmarried	19 (55.9)	15 (44.1)	
**Husband educational status**			
Unable to read and write	15 (25.8)	43 (74.2)	25.772, 0.0001^*^
Able to read and write	17 (34)	33 (66)	
Grade 1–4	16 (66.7)	8 (33.3)	
Grade 5–8	21(50)	21 (50)	
Grade 8–10	12 (37.5)	20 (62.5)	
Grade 11–12	33 (57.9)	24 (42.1)	
Diploma and above	34 (60.7)	22 (39.3)	
**Husband occupation**			
Student	9 (47.4)	10 (52.6)	8.261, 0.082
Government employee	42 (55.3)	34 (44.7)	
Private employee	45 (51.7)	42 (48.3)	
Merchant	35 (41.2)	50 (58.8)	
Farmer	17 (32.7)	35 (67.3)	
**Ethnicity**			
Amhara	162 (48.2)	174 (51.8)	3.201, 0.362
Others^¥¥¥^	5 (29.4)	12 (70.6)	
**Residence**			
Urban	131 (50.8)	127 (49.2)	4.621, 0.032^**^
Rural	36 (37.9)	59 (62.1)	
**Average monthly income**			
< 1,527 ETB	58 (41.7)	81 (58.3)	14.454, 0.002 ^***^
1,527–3,000 ETB	41 (41.4)	58 (58.6)	
3,001–5,305 ETB	35 (50.7)	34 (49.3)	
>5,305 ETB	33 (71.7)	13 (28.3)	

Others^¥^ (Muslim, protestant and catholic), ^¥¥^ (student, daily laborer), ^¥¥¥^ (Oromo, Tigrie, Agew).

### Obstetric-related characteristics

The majority (64.9%) of the current born babies was male, and 49.3% of these male babies had good maternal delivery referral practices. However, 50.7% of them born from women who did not practice good maternal referral. Nearly 94% of mothers who received poor delivery referral practice gave birth to a live fetus, while 96% of mothers who received poor referral treatment experienced stillbirth. With maternal delivery referral practice, there are considerable variations in gestational age and delivery method for the most recent birth. However, there was little correlation between the amount of maternal delivery referral practice and the parity, cause for referral, fetal outcome, ANC follow-up, and history of bleeding before and after birth (Table 2).

**Table 2:** Obstetric-related characteristics of the respondents in public hospitals of Bahir Dar City, Ethiopia, 2021 (*n* = 353).

**Variables**	**Maternal delivery referral practice**		**X^2^, *P*-Value**
	**Good**	**Poor**	
**Parity**			
Primipara	93 (44.7)	115 (55.3)	1.370, 0.242
Multipara	74 (51.03)	71 (48.97)	
**Reason for referral**			
Previous C/S	22 (46.8)	25 (53.2)	7.845, 0.097
Hypertension/PIH	38 (57.6)	28 (42.4)	
Cephalo-pelvic disproportion	31 (38.3)	50 (61.7)	
Abnormal presentation/position	31 (41.3)	44 (58.7)	
Others	45 (53.6)	39 (46.4)	
**The current pregnancy wanted**			
Yes	142 (46.1)	166 (53.9)	1.407, 0.236
No	25 (55.6)	20 (44.4)	
**Gestational age**			
Preterm	58 (40.3)	86 (59.7)	7.282, 0.026^*^
Term	101 (54)	86 (46)	
Post term	8 (36.4)	14 (63.6)	
**Mode of delivery of the current birth**			
Spontaneous vaginal delivery	107 (55.2)	87 (44.8)	12.456, 0.002^**^
Assisted delivery	7 (25.9)	20 (74.1)	
Cesarean delivery	53 (40.2)	79 (59.8)	
**The fetal outcome of the current birth**			
Alive	160 (93.6)	11 (6.4)	0.539, 0.463
Stillbirth	7 (3.8)	175 (96.2)	
**Sex of the current baby**			
Male	113 (49.3)	116 (50.7)	1.084, 0.316
Female	54 (43.5)	70 (56.5)	
**Did you have ANC follow-up of the current birth**			
Yes	150 (46.7)	171 (53.3)	0.478, 0.490
No	17 (53.1)	15 (46.9)	
**Did you face bleeding before the birth of this current baby**			
Yes	30 (42.9)	40 (57.1)	0.694, 0.405
No	137 (48.4)	146 (51.6)	
**Did you face bleeding during delivery or later of this birth**			
Yes	55 (44.7)	68 (55.3)	0.509, 0.475
No	112 (48.7)	118 (51.3)	

### Knowledge of delivered mothers about Ethiopian healthcare referral system

According to the survey, 187 (52.9%) of the respondents had heard about maternal delivery referral systems, and 86 (44.9%) of those respondents cited health personnel as their sources of information. About 77.5, 67.7, 44.5, 42.2, 51.8, and 52.4% of respondents were aware that the maternal delivery referral system in Ethiopia is influenced by factors such as directionality, distance from the facility and between facilities, availability of referral forms, ambulance availability, community involvement, and clients' financial capacity, respectively. Accordingly, 87 (24.6%) of the respondents had sufficient understanding of the Ethiopian healthcare referral system (Table 3).

**Table 3:** Knowledge of delivered mothers about Ethiopian healthcare referral system in public hospitals of Bahir Dar City, Ethiopia, 2021 (*n* = 353).

**Variables**	**Frequency (*f*)**	**Percentage (%)**
Adequate knowledge	87	24.6
Inadequate knowledge	266	75.4
Ever heard about maternal delivery referral system	187	52.9
Directionality affects the referral system in Ethiopian context (*n* = 187)	145	77.5
Distance of facilities from one another can affect the obstetric referral practice	239	67.7
Availability of referral forms/slips and other communication networks can influence the referral system	157	44.5
Availability of ambulance in the referring health facility has a role in the referral system	149	42.2
Social/family support has role to play in the referral system	143	40.5
Preference of the client has a role to play in the referral system	204	57.8
Counseling role of the referral officer could influence the referral system	181	51.3
Community participation is important in referral system	183	51.8
Financial ability of the client is a factor to be considered in the referral system	186	52.4

### Health facility and healthcare access related characteristics

The villages where the mothers who gave birth had functional health development army for maternal delivery referral in about 31.4% of the cases. With a maternal delivery referral practice, there are significant variations in the amount of time spent by mothers to be seen by a health worker, the mode of transportation used during referral of a maternal delivery, the type of the referring health facility, the level of the referring health facility, and the distance from the nearest hospital in kilometers (Table 4).

**Table 4:** Health facility and healthcare access related characteristics and maternal referral practice in public hospitals of Bahir Dar City, Ethiopia, 2021 (*n* = 353).

**Variables**	**Maternal delivery referral practice**		**X^2^, *P*-Value**
	**Good**	**Poor**	
**Distance from nearest hospital in Kms**			
< 5	155 (50.5)	152 (49.5)	4.585, 0.032^*^
≥5	31 (67.4)	15 (32.6)	
**Functional village health developmental army**			
**for maternal delivery referral**			
Present	91 (81.9)	20 (18.1)	2.770, 0.096^*^
Absent	95 (39.3)	147 (60.7)	
**Level of the referring health facility**			
Health center	25 (39.7)	38 (60.3)	12.952, 0.005^****^
Primary hospital	108 (54.8)	89 (45.2)	
General hospital	19 (42.2)	26 (57.8)	
Referral hospital	34 (70.8)	14 (29.2)	
**Type of the referring health facility**			
Public	168 (55.5)	135 (44.5)	13.499, 0.001^*^
Others^¥^	18 (20)	32 (64)	
**Time spent to be seen by a health worker**			
< 10 min	111 (57.5)	82 (42.5)	7.273, 0.026^*^
10–30 min	56 (51.9)	52 (48.1)	
>30 min	19 (36.5)	33 (63.5)	
**Means of transportation during referral of maternal delivery**			
Ambulance	147 (72.1)	57 (27.9)	72.726, 0.0001^*^
Other	39 (26.2)	110 (73.8)	

### Level of maternal delivery referral practice

The level of poor maternal delivery referral practice among women was 52.7% (95% CI: 47, 58) after taking 13 maternal delivery referral practice assessment questions, calculating the composite index value, and dichotomizing the composite value.

### Factors associated with maternal delivery referral practice

The final model for maternal delivery referral practice comprised maternal educational status, residency, monthly income, level of referring health facility, mode of delivery of current birth, and travel time to closest hospital. The multivariable logistic regression analysis revealed that women with unable to read and write (AOR = 2.4, 95%CI: 1.2, 4.9), with able to read and write (AOR = 6.6, 95% CI: 2.5, 7.2); monthly income < 1,527 ETB (AOR = 4.6, 95%CI: 1.9, 10.8), income between 1,527 and 3,000 ETB (AOR = 4.3, 95%CI: 1.8, 10.5), and between 3,001 and 5,305 ETB (AOR = 3.7, 95%CI: 1.5, 9.3); who referred from referral hospital (AOR = 4.6, 95% CI:1.9, 11.1), delivered using assisted delivery (AOR = 4.7, 95% CI: 1.7, 13.9), delivered using cesarean (AOR = 2.1, 95% CI: 1.2, 3.5), and >1 h travel time to nearby hospital (AOR = 2.2, 95% CI:1.1, 4.3) had increased likelihood of being good maternal delivery referral practice (Table 5).

**Table 5:** Factors associated with maternal delivery referral practice in public hospitals of Bahir Dar City, North West Ethiopia, 2021.

**Variable**	**Maternal delivery referral practice**		**COR (95%CI)**	**AOR (95%CI)**
	**Good**	**Poor**		
**Maternal educational status**				
Unable to read and write	37	60	2.5 (1.4, 4.6)	2.4 (1.2, 4.9)^*^
Able to read and write	9	34	5.9 (2.5, 13.9)	6.6(2.5, 7.2)^***^
Grade 1–8	28	22	3.1 (0.5, 17.9)	3.1 (0.5,19.2)
Grade 8–12	45	39	1.2 (0.6, 2.4)	1.2 (0.6, 2.6)
Diploma and above	48	31	1.00	1.00
**Monthly income**				
< 1,527 birr	58	81	3.5 (1.7, 7.3)	4.6 (1.9,10.8)^***^
1,527–3,000 birr	41	58	3.6 (1.7, 7.6)	4.3 (1.8, 10.5)^***^
3,001–5,305 birr	35	34	2.5 (1.1, 5.45)	3.7 (1.5, 9.3)^**^
>5,305-birr	33	13	1.00	1.00
**Level of the referring health facility**				
Health center	38	25	1.00	1.00
Primary hospital	89	108	1.8 (1.0, 3.3)	1.5 (0.8, 2.9)
General hospital	26	19	1.1 (0.5, 2.4)	1.4 (0.6, 3.5)
Referral hospital	14	34	3.7 (1.7, 8.2)	4.6 (1.9, 11.1)^***^
**Mode of delivery of the current birth**				
Spontaneous vaginal delivery	107	87	1.00	1.00
Assisted delivery	7	20	3.5 (1.4, 8.7)	4.7 (1.7, 13.9)^**^
Cesarean delivery	53	79	1.8 (1.2, 2.8)	2.1 (1.2, 3.5)^**^
**Time taken to arrive to nearby hospital**				
< 30 min	120	112	1.00	1.00
30–60 min	29	32	1.2 (0.7, 2.1)	1.0 (0.6, 1.9)
>60 min	18	42	2.5 (1.4, 4.6)	2.2 (1.1, 4.3)^*^

^*^ Significant at p < 0.05, ^**^ significant at P < 0.01, ^***^ significant at P < 0.00.

## Discussion

To our knowledge is concerned, this is the first study assessing referral practices among delivered mothers. The study confirmed that the poor maternal delivery referral practice could be higher in magnitude in the variable of interest of most socio-demographic and economic factors, obstetrics, over all knowledge about referral system and health facility and characteristics related to health access. However, the majority of obstetric traits and skills had no relationship with maternal delivery referral practice and were left out of the final regression model.

The WHO has designed a framework or one of the standards known as the referral system, which specifies that all women with any conditions that cannot be adequately treated with the resources available be appropriately referred. This has been done to improve the quality of care for mothers at time of delivery (21). However, compared to a study done in Tanzania, the proportion of poor maternal referral practices in the current study was 52.7% (95% CI: 47%, 58%; 3). The differences in the state of maternal health system practices that may be identified in various nations, such as the policy of maternal referral systems and different measuring instruments, may be the possible explanation for this greater level of bad maternal referral practices. The various sample size estimates, the study's context, and sociocultural norms were also likely contributors to this degree of variation. Similar to other studies, the current study was carried out in the regional capital city of the Amhara state referral hospitals, which include tertiary and specialized comprehensive hospitals with a large number of different patient flows that deteriorate the standard of the maternal referral system (22, 23). The referral healthcare facility's lower level of quality of care is another explanation for the low compliance of mothers to adhere to the referral system (22, 23). In this study, mothers who were referred from lower-level facilities, including health centers had poor referral practices for maternal delivery, consistent with other studies (24). Policymakers must therefore focus on capacitating the referral tier system not only in the upper level of health system but also strongly give emphasis in the lower levels of healthcare wings.

The knowledge, skills, and educational experiences of healthcare professionals, as well as the maternal educational status, which is characterized by late referrals, have a significant impact on the referral system in general and the maternal referral system in particular (24–27). In line with other study, the current study found an association between maternal educational status and maternal delivery referral patterns (26). Some mothers may have believed that all levels of further care provided were in the same practices of care as those of the lower level of care due to their lower educational enrollment, which is one of the misconceptions that stifled good delivery referral practices, especially in resource-constrained settings like Ethiopia. The hierarchy, capabilities, and restrictions of each healthcare institution, as well as the need for referral, were not understood by the laboring women. Therefore, the governments should take an assignment on implementing educational interventions that focus on the women who are in the reproductive age range with little literacy coverage. Moreover, health professionals must also undergo continuous professional development in order to identify complications during any stage of labor, manage them, and quickly refer women during delivery.

The fact that physical accessibility and distance to healthcare settings are the primary reasons why mothers did not adhere to the referral system in many resource-limited settings (28–31) is similar to the findings of the current study, which found that longer travel times to nearby higher level healthcare facilities caused delivered mothers to poorly adhere to the referral linkage. This may be explained by the fact that longer travel times for newly delivered mothers to the next level of care resulted in both direct and indirect costs for food, transportation, and production day loss. This is the most common phenomenon that explains why mothers in low-income nations like Ethiopia did not accept the recommendations of healthcare professionals and prefer to seek care from traditional healthcare providers at home rather than seeking more advanced care. Another studies have also confirmed that one of the three delays that contribute to maternal deaths is a delay in transportation (28, 31, 32).

Also, one of the obstacles preventing most mothers from following the referral system to higher level healthcare institutions during labor was the prevalent false perception of assisted delivery, such as cesarean section (24). A significant percentage of mothers connected the referral system to needing a cesarean delivery (4, 29–31). In a similar vein, the current study found a strong correlation between poor maternal referral practices and cesarean birth. Most often, assisted and cesarean section deliveries require a swift decision and skill in handling emergency procedures, but most Ethiopian professionals, particularly those working in the lower levels of the health system, are not well equipped to handle such services, which is why maternal referrals are delayed in the absence of an adequate facility for such a procedure. On the other hand, childbirth professionals irresponsibly referred the mothers without paying enough attention to the referral processes, which were characterized by confusion, dilemmas, and dread of problems after the mothers had already exhausted. Additionally, avoiding responsibility in the event that the outcome of the mother's delivery would be poor led to poor maternal referral practices. Therefore, the ministry of health and women should reinforce the legal system's due process and protection of women's rights.

Given that most government facilities in Ethiopia do not charge for maternal healthcare-related services, and that motherhood income is a known impediment to referral systems (20, 32–34), the study found that poor maternal delivery referral practices were significantly associated with low monthly income. One social factor of health, poverty, made it more likely for women to have babies with poor maternal referral practices and birth outcomes. This level of referral practice might be exacerbated mainly the weak functionality of the ambulance services and free of indirect medical costs. In the lines share of rural health facilitates, ambulance services were politicized and not actively serving the intended users such as mothers which intern forced them to use public transport out-of-pocket which is neither fast, sheep or healthy. The poor efficiency of the ambulance services may be also the main cause of this poor referral practice. This study had certain constraints, of course. Since the study was cross-sectional, it is impossible to conclude causation because the components do not show a temporal link. The study's findings might only be applicable to those mothers with history of healthcare facility attendance for delivery. The most prevalent bias was recall bias. To lessen the impact of this bias, probing techniques were used. It can also be challenging to gather self-reported data on sensitive topics like motherhood income because these topics can be extremely private and frequently relate to self-image and personality.

## Conclusion

Maternal delivery referral practices were generally found to be poor and delivery referral standards are not adequately complied because of the fact that low levels of motherhood income, low educational attainment, lack of transportation options, and a longer travel time to a higher-level healthcare facilities. The healthcare accessibility and mode of delivery were also belonged to those important factors that independently influenced maternal delivery referral practice. Maternal delivery referral practice could be improved through mass maternal education. In order to ensure that services were more client-centered; the study strongly suggested that greater efforts has to be made in constructing facilities close to the direct points of use by mothers. Low-cost transport is also needed to mitigate barriers to referral by availing ambulance. To ensure quality maternal referrals, district-level health managers should be trained and equipped with the skills needed to monitor and evaluate referral documentation, including the quality and efficiency of maternal referrals. It is also important to conduct additional qualitative research on maternal referrals, namely concentrating on the implications of policy in improving the referral tier system.

## Data Availability

The raw data supporting the conclusions of this article will be made available by the authors, without undue reservation.

## References

[1] World Health Organization. The Network for Improving Quality of Care for Maternal, Newborn and Child Health: Evolution, Implementation and Progress: 2017–2020 Report. (2021). Available at: https://apps.who.int/iris/bitstream/handle/10665/343370/9789240023741-eng.pdf?sequence=1 (accessed October 11, 2011).

[2] Kim-Hwang JE Chen AH Bell DS Guzman D Yee HF Kushel MB. Evaluating electronic referrals for specialty care at a public hospital. J Gen Intern Med. (2010) 25:1123–8. 10.1007/s11606-010-1402-1 20512531 PMC2955477

[3] Font F Quinto L Masanja H Nathan R Ascaso C Menendez C. Paediatric referrals in rural Tanzania: the Kilombero District study—a case series. BMC Int Health Hum Rights. (2002) 2:4. 10.1186/1472-698X-2-4 11983024 PMC111197

[4] Manu A Hill Z Ten Asbroek AH Soremekun S Weobong B Gyan T. Increasing access to care for sick newborns: evidence from the Ghana Newhints cluster-randomised controlled trial. BMJ Open. (2016) 6:e008107. 10.1136/bmjopen-2015-008107 27297006 PMC4916576

[5] Kenya service provision assessment survey 2010. Nairobi: - Google Scholar. Available at: https://scholar.google.com/scholar?hl=en&as_sdt=0%2C5&q=Kenya+service+provision+assessment+survey+2010.+Nairobi%3A+National+Coordinating+Agency+for+Population+and+Development%2C+Ministry+of+Medical+Services%2C+Ministry+of+Public+Health+and+Sanitation%2C+Kenya+National+Bureau+of+Statistics%2C+and+ICF+Macro.&btnG= (accessed October 11, 2021).

[6] Rehman T Keepanasseril A Maurya DK Kar SS. Factors associated with maternal referral system in South India: a hospital-based cross-sectional analytical study. J Nat Sc Biol Med. (2020) 11:158–63.

[7] Diba F Ichsan I Muhsin M Marthoenis M Marthoenis M Sofyan H. Healthcare providers' perception of the referral system in maternal care facilities in Aceh, Indonesia: a cross-sectional study. BMJ Open. (2019) 9:e031484. 10.1136/bmjopen-2019-031484 31818837 PMC6924809

[8] Rudge MVC Maestá I Moura PMSS Rudge CVC Morceli G Costa RAA. The safe motherhood referral system to reduce cesarean sections and perinatal mortality—a cross-sectional study [1995–2006]. Reprod Health. (2011) 8:34. 10.1186/1742-4755-8-34 22108042 PMC3256099

[9] Tayler-Smith K Zachariah R Manzi M Van den Boogaard W Nyandwi G Reid T. An ambulance referral network improves access to emergency obstetric and neonatal care in a district of rural Burundi with high maternal mortality. Trop Med Int Health. (2013) 18:993–1001. 10.1111/tmi.12121 23682859

[10] Accorsi S Somigliana E Solomon H Ademe T Woldegebriel J Almaz B. Cost-effectiveness of an ambulance-based referral system for emergency obstetrical and neonatal care in rural Ethiopia. BMC Pregnancy Childbirth. (2017) 17:220. 10.1186/s12884-017-1403-8 28701153 PMC5506594

[11] Girma M Yaya Y Gebrehanna E Berhane Y Lindtjørn B. Lifesaving emergency obstetric services are inadequate in south-west Ethiopia: a formidable challenge to reducing maternal mortality in Ethiopia. BMC Health Serv Res. (2013) 13:459. 10.1186/1472-6963-13-459 24180672 PMC4228432

[12] Magge H Kiflie A Nimako K Brooks K Sodzi-Tettey S Mobisson-Etuk N. The Ethiopia healthcare quality initiative: design and initial lessons learned. Int J Qual Health Care. 31:G180–6. 10.1093/intqhc/mzz127 31834384

[13] Mirkuzie AH Sisay MM Bedane MM. High proportions of obstetric referrals in Addis Ababa: the case of term premature rupture of membranes emergency medicine. BMC Res Notes. (2016) 9:40. 10.1186/s13104-016-1852-6 26809734 PMC4724955

[14] Sharma A Kumari A Kumari A Chauhan A Ankita Anchal. An experimental study to compare the effectiveness of hot application versus tapping therapy among pregnant women admitted in maternity ward with labor pain in selected hospitals of Ambala/Haryana. Int J Midwifery Nurs. (2018) 1:205–18.

[15] Bailey PE Keyes EB Parker C Abdullah M Kebede H Freedman L. Using a GIS to model interventions to strengthen the emergency referral system for maternal and newborn health in Ethiopia. Int J Gynecol Obstet. (2011) 115:300–9. 10.1016/j.ijgo.2011.09.004 21982854

[16] NASR E Raeisi P Motlagh M Kabir M. Evaluation of the Performance of Referral System in Family Physician Program in Iran University of Medical Sciences: 2009. (2010). Available at: https://www.sid.ir/en/journal/ViewPaper.aspx?id=185960 (accessed October 11, 2021).

[17] Jandorf L Cooperman J Stossel LM Itzkowitz S Thompson HS Villagra C. Implementation of culturally targeted patient navigation system for screening colonoscopy in a direct referral system. Health Educ Res. (2013) 28:803–15. 10.1093/her/cyt003 23393099 PMC3772329

[18] Pembe AB Carlstedt A Urassa DP Lindmark G Nyström L Darj E. Effectiveness of maternal referral system in a rural setting: a case study from Rufiji district, Tanzania. BMC Health Serv Res. (2010) 10:326. 10.1186/1472-6963-10-326 21129178 PMC3003655

[19] Rasoulinezhad S Rasoolinejad M. A Study of Referral System in Health Care Delivery System and Recommended Alternative Strategies: Kashan Health Care. (2002). Available at: https://www.sid.ir/en/Journal/ViewPaper.aspx?ID=48004 (accessed October 11, 2021).

[20] Kananura RM Kiwanuka SN Ekirapa-Kiracho E Waiswa P. Persisting demand and supply gap for maternal and newborn care in eastern Uganda: a mixed-method cross-sectional study. Reprod Health. (2017) 14:136. 10.1186/s12978-017-0402-6 29065922 PMC5655951

[21] Devarbhavi P Telang L Vastrad B Tengli A Vastrad C Kotturshetti I. Identification of key pathways and genes in polycystic ovary syndrome via integrated bioinformatics analysis and prediction of small therapeutic molecules. Reprod Biol Endocrinol. (2021) 19:31. 10.1186/s12958-021-00706-3 33622336 PMC7901211

[22] Chaturvedi S Randive B Diwan V De Costa A. Quality of obstetric referral services in India's JSY cash transfer programme for institutional births: a study from madhya pradesh province. PLoS ONE. (2014) 9:e96773. 10.1371/journal.pone.0096773 24810416 PMC4014551

[23] Afari H Hirschhorn L Michaelis A Barker P Sodzi-Tettey S. Quality improvement in emergency obstetric referrals: qualitative study of provider perspectives in Assin North district, Ghana. BMJ Open. (2014) 45:e005052. 10.1136/bmjopen-2014-005052 24833695 PMC4025473

[24] Josyula S Taylor KK Murphy BM Rodas D Kamath-Rayne BD. Obstetric referrals from a rural clinic to a community hospital in Honduras. Midwifery. (2015) 31:1054–9. 10.1016/j.midw.2015.07.002 26228586

[25] Windsma M Vermeiden T Braat F Tsegaye AM Gaym A Van Den Akker T. Emergency obstetric care provision in Southern Ethiopia: a facility-based survey. BMJ Open. (2017) 7:e018459. 10.1136/bmjopen-2017-018459 29122802 PMC5695514

[26] Elmusharaf K Byrne E AbuAgla A AbdelRahim A Manandhar M Sondorp E. Patterns and determinants of pathways to reach comprehensive emergency obstetric and neonatal care (CEmONC) in South Sudan: qualitative diagrammatic pathway analysis. BMC Pregnancy Childbirth. (2017) 17:278. 10.1186/s12884-017-1463-9 28851308 PMC5576292

[27] Maranto M Gullo G Bruno A Minutolo G Cucinella G Maiorana A. Factors associated with anti-SARS-CoV-2 vaccine acceptance among pregnant women: data from outpatient women experiencing high-risk pregnancy. Vaccines. (2023) 11:454. 10.3390/vaccines11020454 36851330 PMC9966581

[28] Greenspan JA Chebet JJ Mpembeni R Mosha I Mpunga M Winch PJ. Men's roles in care seeking for maternal and newborn health: a qualitative study applying the three delays model to male involvement in Morogoro Region, Tanzania. BMC Pregnancy Childbirth. (2019) 19:293. 10.1186/s12884-019-2439-8 31409278 PMC6693212

[29] Nakano K Nakamura Y Shimizu A Alamer SM. Exploring roles and capacity development of village midwives in Sudanese communities. Rural Remote Health. (2018) 18:4668. 10.22605/RRH4668 30343581

[30] Long Q Madede T Parkkali S Chavane L Sundby J Hemminki E. Maternity care system in Maputo, Mozambique: plans and practice? Cogent Med. (2017) 4:1412138. 10.1080/2331205X.2017.1412138

[31] Zaami S Montanari Vergallo G Napoletano S Signore F Marinelli E. The issue of delivery room infections in the Italian law. A brief comparative study with English and French jurisprudence. J Matern Neonatal Med. (2018) 31:223–7. 10.1080/14767058.2017.1281243 28076992

[32] Pembe AB Mbekenga CK Olsson P Darj E. Why do women not adhere to advice on maternal referral in rural Tanzania? Narratives of women and their family members. Glob Health Action. (2017) 10:1364888. 10.1080/16549716.2017.1364888 28856975 PMC5645683

[33] Brooks MI Thabrany H Fox MP Wirtz VJ Feeley FG Sabin LL. Health facility and skilled birth deliveries among poor women with Jamkesmas health insurance in Indonesia: a mixed-methods study. BMC Health Serv Res. (2017) 17:1–12. 10.1186/s12913-017-2028-3 28148258 PMC5288898

[34] Al-Adhroey AH Mehrass AA-KO Al-Shammakh AA Ali AD Akabat MYM Al-Mekhlafi HM. Prevalence and predictors of Toxoplasma gondii infection in pregnant women from Dhamar, Yemen. BMC Infect Dis. (2019) 19:1089. 10.1186/s12879-019-4718-4 31888517 PMC6937662

